# In-house chromogenic anti-factor Xa assay: development, validation, and identification of factors predicting APTT discordance

**DOI:** 10.3389/fmed.2025.1745447

**Published:** 2026-01-12

**Authors:** Lin Sun, Jinxia Zhao, Guohong Jiang, Wenfei Lu, Hui Rong, Limin Lun, Yuehan Wang, Tianhui Zhou

**Affiliations:** 1 Department of Laboratory Medicine, The Affiliated Hospital of Qingdao University, Qingdao, China; 2 Laboratory Department, Qingdao Public Health Clinical Center, Qingdao, China; 3 Beijing ZONCI Technology Development Co., Ltd., Beijing, China

**Keywords:** activated partial thromboplastin time, anticoagulation monitoring, classification discordance, predictive modeling, unfractionated heparin

## Abstract

**Background:**

Activated partial thromboplastin time (APTT) is the conventional test for monitoring unfractionated heparin (UFH) therapy, but discordance with anti-factor Xa results frequently occurs in clinical practice, potentially leading to suboptimal anticoagulation management.

**Objective:**

This study aimed to develop an in-house anti-factor Xa detection system, establish laboratory-specific APTT therapeutic ranges calibrated against anti-factor Xa levels, and identify predictors of discordance between these two monitoring methods.

**Methods:**

We developed and validated a chromogenic anti-factor Xa assay according to CLSI guidelines. From 110 patients receiving UFH, 190 samples were collected. Laboratory-specific APTT ranges were derived using linear regression and ROC analysis against anti-factor Xa target range (0.3–0.7 IU/ml). Random forest and logistic regression models identified predictors of discordance among clinical and laboratory variables.

**Results:**

The optimized assay showed excellent analytical performance with strong correlation to the commercial platform (Pearson *r* = 0.986, *R*^2^ = 0.972). The derived laboratory-specific APTT therapeutic range was 79–127 s. Despite moderate correlation between APTT and anti-factor Xa (Spearman ρ = 0.678, *p* < 0.001), classification concordance was poor, with 58.2% of specimens showing discordance. Random forest analysis (AUC = 0.79) and logistic regression identified five key predictors: fibrinogen, triglycerides, estimated glomerular filtration rate, C-reactive protein, and antithrombin III.

**Conclusions:**

This study developed a validated anti-factor Xa assay and identified substantial classification discordance between APTT and anti-factor Xa monitoring. Five clinical and laboratory factors were associated with monitoring discordance.

## 1 Introduction

Unfractionated heparin (UFH) is widely used for anticoagulation in acute coronary syndromes, venous thromboembolism, cardiac surgery, and hemodialysis. UFH binds to antithrombin III (AT-III), thereby accelerating the inactivation of thrombin and factor Xa (1). However, the anticoagulant response to UFH demonstrates considerable inter-individual and intra-individual variability, influenced by multiple factors including plasma protein binding, renal clearance, inflammatory status, and antithrombin levels (2–4), necessitating therapeutic drug monitoring (5). However, as a global coagulation screening test, APTT is susceptible to interference from numerous pre-analytical and analytical variables. Endogenous coagulation factor levels (particularly factors VIII and XII), fibrinogen concentration, lupus anticoagulants, and variable heparin sensitivity of different APTT reagents can all affect test results (6–9). These limitations compromise inter-laboratory comparability and may lead to subtherapeutic or supratherapeutic anticoagulation states, increasing the risk of thrombotic recurrence or hemorrhagic complications (10).

Chromogenic substrate-based anti-factor Xa assays directly quantify the inhibitory activity of the heparin-antithrombin complex against factor Xa, offering improved specificity compared to APTT (11, 12). Chromogenic anti-factor Xa assays measure heparin activity by incubating patient plasma with excess factor Xa; the heparin-antithrombin complex neutralizes factor Xa, and residual enzyme activity is quantified via chromogenic substrate cleavage, providing direct measurement of anticoagulant effect. However, the high cost of commercially available anti-factor Xa platforms has limited their widespread adoption in many clinical laboratories (13–15), creating a need for more accessible testing options.

Discordance in anticoagulation status classification (subtherapeutic, therapeutic, or supratherapeutic) between APTT and anti-factor Xa results is commonly observed, with reported inconsistency rates of 30%−50% (16, 17). While several studies identified potential contributing factors—antithrombin deficiency, elevated factor VIII, acute phase reactions, renal dysfunction (18–20)—a comprehensive predictive model integrating multiple clinical and laboratory variables has not been established.

Traditional statistical approaches may not fully capture the complex, nonlinear interactions among multiple predictors of assay discordance. Machine learning algorithms, particularly random forest (RF) models, can handle high-dimensional data, identify nonlinear relationships, and quantify variable importance without assuming parametric distributions (14, 21). Recent applications of RF in coagulation research have demonstrated its utility in predicting bleeding risk and thrombotic complications. Recent applications of machine learning in coagulation research have demonstrated utility in predicting bleeding risk (22), venous thromboembolism in cancer patients (23), and optimizing anticoagulant dosing (24). However, to our knowledge, no study has applied machine learning methods to identify predictors of APTT-anti-factor Xa discordance in UFH monitoring.

This study has three primary objectives: first, to develop an in-house chromogenic anti-factor Xa assay. Second, to establish a laboratory-specific APTT therapeutic range calibrated against the anti-factor Xa target (0.3–0.7 IU/ml) (25, 26). Third, to identify predictors of APTT-anti-factor Xa discordance using random forest and logistic regression approaches.

## 2 Materials and methods

### 2.1 Study design and sample collection

#### 2.1.1 Ethical approval

This study was approved by the Ethics Committee of the Affiliated Hospital of Qingdao University (Approval No. QYFY WZLL 30726). Written informed consent was obtained from all participants. All data were anonymized per Declaration of Helsinki principles.

#### 2.1.2 Study population

Between March 2024 and September 2024, 190 blood samples were prospectively collected from 110 consecutively enrolled hospitalized patients receiving continuous intravenous UFH at the Affiliated Hospital of Qingdao University. UFH indications included acute coronary syndrome (*n* = 38, 34.5%), VTE treatment/prophylaxis (*n* = 24, 21.8%), post-cardiac surgery (*n* = 15, 13.6%), hemodialysis/CRRT (*n* = 8, 7.3%), and other indications (*n* = 25, 22.7%).

Inclusion criteria: age ≥18 years; receiving intravenous UFH ≥24 h; both APTT and anti-factor Xa testing conducted within 4 h of blood collection.

Exclusion criteria: concurrent treatment with direct oral anticoagulants, low molecular weight heparin, or vitamin K antagonists; severe liver dysfunction (Child-Pugh class C); known inherited coagulation disorders; hemolyzed, icteric, or severely lipemic specimens (triglycerides >11.3 mmol/L); incomplete laboratory data.

Blood sampling was conducted 3–6 h after UFH bolus or during steady-state infusion (continuous intravenous UFH administration ≥24 h at constant rate; samples collected ≥6 h after last dose adjustment) (5, 26).

#### 2.1.3 Blood collection and processing

We collected blood via clean venipuncture into 3.2% trisodium citrate tubes (9:1 blood:anticoagulant ratio). We obtained Platelet-poor plasma (PPP) by centrifugation at 2,000 × g for 15 min at 15–20 °C. PPP was immediately aliquoted and stored at −80 °C.

#### 2.1.4 Normal pooled plasma preparation

We prepared Normal pooled plasma (NPP) from 280 healthy volunteers with normal coagulation parameters. Individual PPP were pooled, aliquoted, and frozen at −80 °C.

#### 2.1.5 Sample allocation

The 190 specimens were allocated as follows:

- APTT therapeutic range derivation: 80 specimens (from 80 different patients).
- Concordance evaluation and predictive modeling: 110 specimens (from 110 different patients, independent from derivation set).

All specimens were stored at −80 °C and analyzed within 3 months. Frozen aliquots were thawed at 37 °C for 3 min, equilibrated to room temperature for 2 min, and tested within 4 h. Samples with visible fibrin strands were excluded.

### 2.2 Instruments and reagents

#### 2.2.1 Instruments and allocation

XL-3200c Automated Blood Coagulation Analyzer (ZONCI, Beijing, China): in-house anti-Xa assay;STA-Compact R-Max Analyzer (Stago, France): commercial anti-Xa, APTT, fibrinogen, and antithrombin III assays;Roche Cobas c702 (Switzerland): biochemical parameters (CRP, TG, TC, creatinine for eGFR calculation).

#### 2.2.2 In-house anti-Xa assay reagents

Bovine factor Xa (≥95% purity; Aglycons, China); S-2732 chromogenic substrate (Aglyco, China); BSA, Tris, NaCl, HCl, NaOH, PEG-6000 (China National Medicines Corporation Ltd).

#### 2.2.3 Commercial coagulation reagents (all from Stago)

STA^2^®^^-Liquid Anti-Xa reagent, calibrators (0–1.50 IU/ml), and controls; STA^2^®^^-APTT 5 (REF00595); STA^2^®^^-Fibrinogen 5; STA^2^®^^-Antithrombin III.

Representative lot numbers from the final validation phase are provided; multiple lots were used sequentially during the 7-month study period (March–September 2024). Complete lot documentation is available upon request.

### 2.3 Development of the in-house anti-factor Xa activity detection system

#### 2.3.1 Assay principle

The assay employs a two-stage chromogenic method. Patient plasma containing heparin is incubated with excess bovine factor Xa (R1). Heparin-AT-III complex neutralizes added factor Xa. Residual factor Xa cleaves chromogenic substrate S-2732 (R2), releasing para-nitroaniline measured at 405 nm. The rate is inversely proportional to heparin concentration.

#### 2.3.2 Reagent composition optimization

Factor Xa (0.5–4.0 IU/ml), S-2732 (0.3–2.0 mmol/L), buffer system (Tris-HCl 10–30 mmol/L combined with NaCl 0.1%−0.9%, pH range 7.4–8.8), and stabilizers (BSA 0.1%−0.5% w/v for R1; PEG-6000 0.1%−0.5% w/v for R2) were systematically optimized. Sample-to-reagent volume ratios and reaction times were optimized for sensitivity and linear range.

### 2.4 Analytical performance validation

All validation studies followed CLSI guidelines (27).

#### 2.4.1 Precision

We measured three QC levels (low: 0.30 IU/ml; medium: 0.70 IU/ml; high: 1.35 IU/ml) 20 times within-run and in duplicate twice daily over 20 days for Inter-assay precision. Acceptance: within-run CV < 5%, between-run CV < 10%.

#### 2.4.2 Analytical sensitivity

Limit of blank (LoB): NPP (anti-Xa < 0.01 IU/ml) was measured 60 times. LoB = Mean blank + 1.645 × SD blank or the non-parametric method (LoB = 95th percentile of blank measurements).

Limit of detection (LoD): low-concentration samples were measured 60 times. LoD = LoB + 1.645 × SD low sample.

Limit of quantitation (LoQ): three candidate concentrations (0.04, 0.08, 0.10 IU/ml) were evaluated with 20 replicates each using three criteria:

- Precision: relative median absolute deviation (MAD) ≤ 30%

° MAD =median (|x_i_ – median (x)|)
° Relative MAD (%) = (MAD/median) × 100
° Rationale: MAD is more robust to outliers than CV at low concentrations (28, 29).

- Probability of detection (POD): ≥95% of replicates above LoD
- Total error (TE): ≤ 40%

° TE = |Bias| + 2 × SD
° Bias = [(Mean measured – Target)/Target] × 100%

The lowest concentration meeting all three criteria was designated as LoQ.

#### 2.4.3 Linearity

Linearity was evaluated using high-concentration and low-concentration plasma samples according to CLSI guideline EP06 (30). Samples were mixed in different proportions using sample diluent (7H + L, 6H + 2L, 5H + 3L, 4H + 4L, 3H + 5L, 2H + 6L, and H + 7L) to prepare seven dilution levels spanning the concentration range from LoQ to 1.35 IU/ml. Each dilution was measured in duplicate. Linear regression analysis was employed to compare measured values against expected values, and scatter plots were generated.

#### 2.4.4 Method comparison

Eighty anonymized plasma specimens from routine diagnostic testing were tested by both in-house and Stago systems. Pearson or Spearman correlation analysis and Bland–Altman agreement analysis were employed to evaluate correlation and concordance between the two systems. Paired *t*-test or Wilcoxon signed-rank test was used to assess systematic differences between methods. Regression equations and coefficients of determination (*R*^2^) were determined. The method comparison protocol followed CLSI guideline EP09c (31).

### 2.5 APTT therapeutic range establishment

#### 2.5.1 Data allocation strategy

Our laboratory's APTT reference range is 28–43.5 s (mean 36 s). The traditional empirical method calculates therapeutic range as 1.5–2.5 × the mean normal value (54–90 s). The 190 specimens from 110 patients were randomly allocated to two distinct datasets using the random number generator in SPSS 27.0 software:

- APTT range derivation (*n* = 80 samples, 42.1%): a randomly selected subset was used to establish laboratory-specific APTT therapeutic ranges through linear regression (APTT = a + b × anti-Xa) and ROC curve analysis. APTT cutoffs corresponding to anti-Xa levels of 0.3 and 0.7 IU/ml were identified.
- Concordance evaluation and predictive modeling (*n* = 110 samples, 57.9%): the remaining independent samples (not included in derivation set) were used for: (a) evaluating APTT-anti-Xa classification concordance; and (b) identifying predictors of discordance using patient-level clinical data.

This allocation strategy ensured independent validation of the derived APTT range while maximizing statistical power for subsequent analyses.

#### 2.5.2 ROC analysis

Two ROC analyses identified optimal APTT cutoffs:

Lower threshold (0.3 IU/ml): samples dichotomized as anti-Xa < 0.3 (subtherapeutic) vs. ≥0.3 IU/ml (therapeutic/above). Optimal APTT cutoff determined by Youden Index maximization.

Upper threshold (0.7 IU/ml): samples dichotomized as anti-Xa ≤ 0.7 (therapeutic/below) vs. >0.7 IU/ml (supratherapeutic). Optimal APTT cutoff determined by Youden Index (32).

#### 2.5.3 Concordance analysis

Using established ranges, each specimen was classified as subtherapeutic, therapeutic, or supratherapeutic. Concordance was defined as agreement between APTT and anti-Xa across all three categories. Overall agreement rate and Cohen's kappa (κ) were calculated.

### 2.6 Predictive modeling

#### 2.6.1 Study design

Given poor concordance between APTT and anti-Xa, we developed predictive models to identify patients at risk for discordance.

Critical limitation: exploratory, hypothesis-generating analysis with overfitting risk. Sample size *n* = 110 with 64 discordant events and 10 predictors yields EPV ≈ 6.4, below recommended minimum of 10. Five-fold cross-validation employed; all findings require external validation.

#### 2.6.2 Variable collection

Ten candidate predictor variables were collected:

- Demographic (3): age, sex, body mass index (BMI)
- Coagulation (3): fibrinogen, AT-III, D-dimer
- Inflammatory (1): C-reactive protein
- Renal (1): estimated glomerular filtration rate (eGFR), calculated using the Chronic Kidney Disease Epidemiology Collaboration equation
- Lipid (2): triglycerides, total cholesterol.

All parameters were measured within 24 h of anticoagulation monitoring.

#### 2.6.3 Statistical modeling

Outcome variable: concordant (0) vs. discordant (1) APTT-anti-Xa classification.

Random forest model: RF model, with bootstrap sampling and class balancing was constructed. Variable importance was assessed by Mean Decrease in Accuracy. Out-of-bag samples provided internal validation. Performance metrics: AUC, sensitivity, specificity, confusion matrix.

Logistic regression model: all 10 variables were entered into multivariable logistic regression. Backward elimination (*p* ≥ 0.05) was *conducted*, retaining clinically important variables (AT-III, fibrinogen) regardless of significance. Multicollinearity (VIF >5), influential observations (Cook's distance >4/*n*), goodness-of-fit (Hosmer–Lemeshow test), and model calibration were assessed. Five-fold cross-validation was conducted.

#### 2.6.4 Statistical analysis

Normally distributed continuous variables: mean ± SD; non-normally distributed: median (IQR); categorical: frequency (%). Concordant vs. discordant groups compared using *t*-test/Mann-Whitney *U*-test (continuous) or chi-square/Fisher's eXact test (categorical). Bonferroni correction applied for multiple comparisons (adjusted α = 0.05/10 = 0.005). Two-tailed *p* < 0.05 considered significant.

Software: SPSS 27.0 (IBM, USA); R 4.2.0 (R Foundation, Austria); MedCalc 20.0 (Belgium).

## 3 Results

### 3.1 Development and optimization of anti-factor Xa assay

#### 3.1.1 Reagent component optimization

Through systematic concentration gradient experiments, optimal reagent compositions were established. Reagent 1 (R1) contained bovine factor Xa at 0.9 IU/ml (selected from 0.8 to 1.0 IU/ml range), while Reagent 2 (R2) contained chromogenic substrate S-2732 at 0.43 mmol/L (selected from 0.4 to 0.5 mmol/L range). This optimized concentration combination yielded excellent linearity (*R*^2^ = 0.9998) for diluted standard plasma.

The buffer system, comprising Tris-HCl (10–30 mmol/L) and NaCl (0.1%−0.9%), was optimized to achieve optimal detection sensitivity and linearity. BSA (0.1%−0.5% w/v) was incorporated into R1 and the sample diluent as a stabilizer, whereas PEG-6000 (0.1%−0.5% w/v) was added to R2 to prevent reagent denaturation without affecting assay performance.

The optimal sample-to-reagent ratio was determined to be 5 μl (sample):40 μl (R1):100 μl (R2). Under these conditions, linear regression analysis of the 7-point calibration curve using diluted standard plasma yielded *R*^2^ = 0.9998, while the correlation between measured and expected values in clinical specimens demonstrated *R*^2^ = 0.9996.

#### 3.1.2 Reaction time optimization

**Figure 1:**
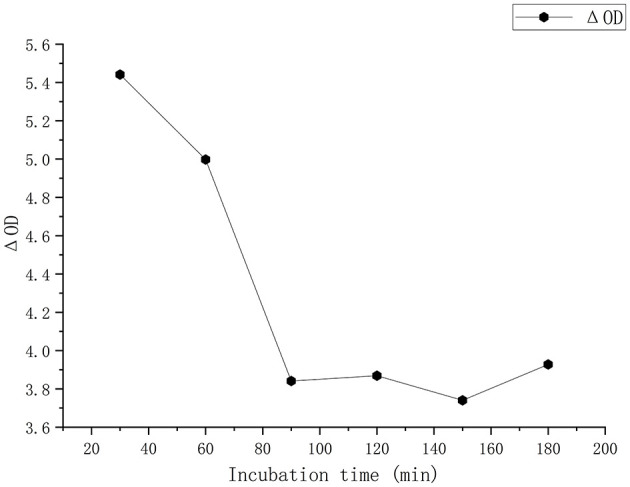
Reaction time optimization. Incubation time optimization showing Δ(OD) decreased rapidly within 60 s and reached equilibrium after 90 s.

Optimal incubation time was 90 s, at which residual heparin in plasma bound maximally to reagent factor Xa, yielding the highest chromogenic signal (Figure 1).

**Figure 2:**
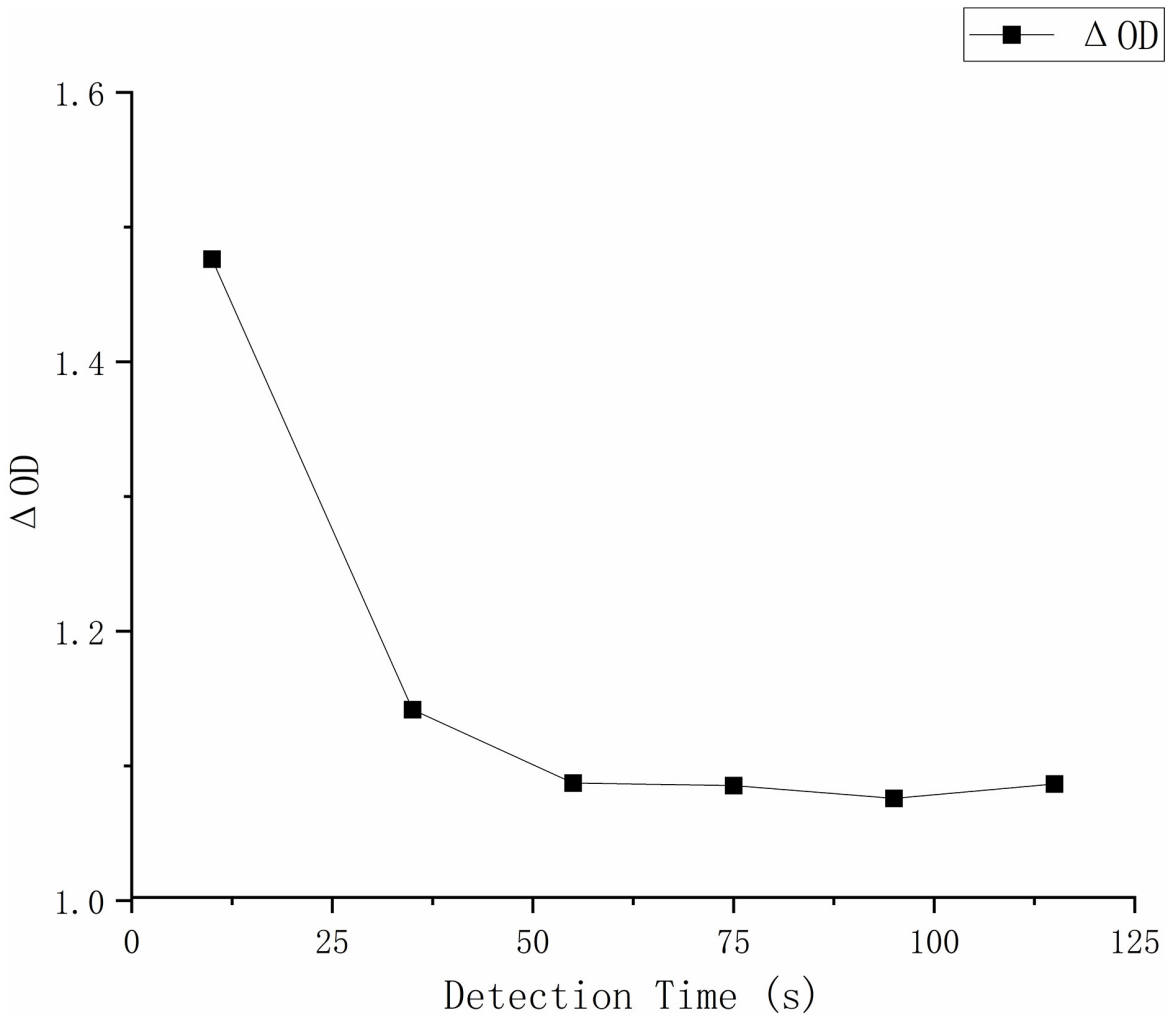
Reaction time optimization. Reaction time optimization. Detection time kinetics showing Δ(OD) declined sharply in the first 25 s and stabilized after 55 s.

During continuous monitoring from 5 to 55 s, the chromogenic reaction reached a plateau phase, after which ΔOD exhibited no further significant changes (Figure 2).

#### 3.1.3 Calibration curves

**Figure 3:**
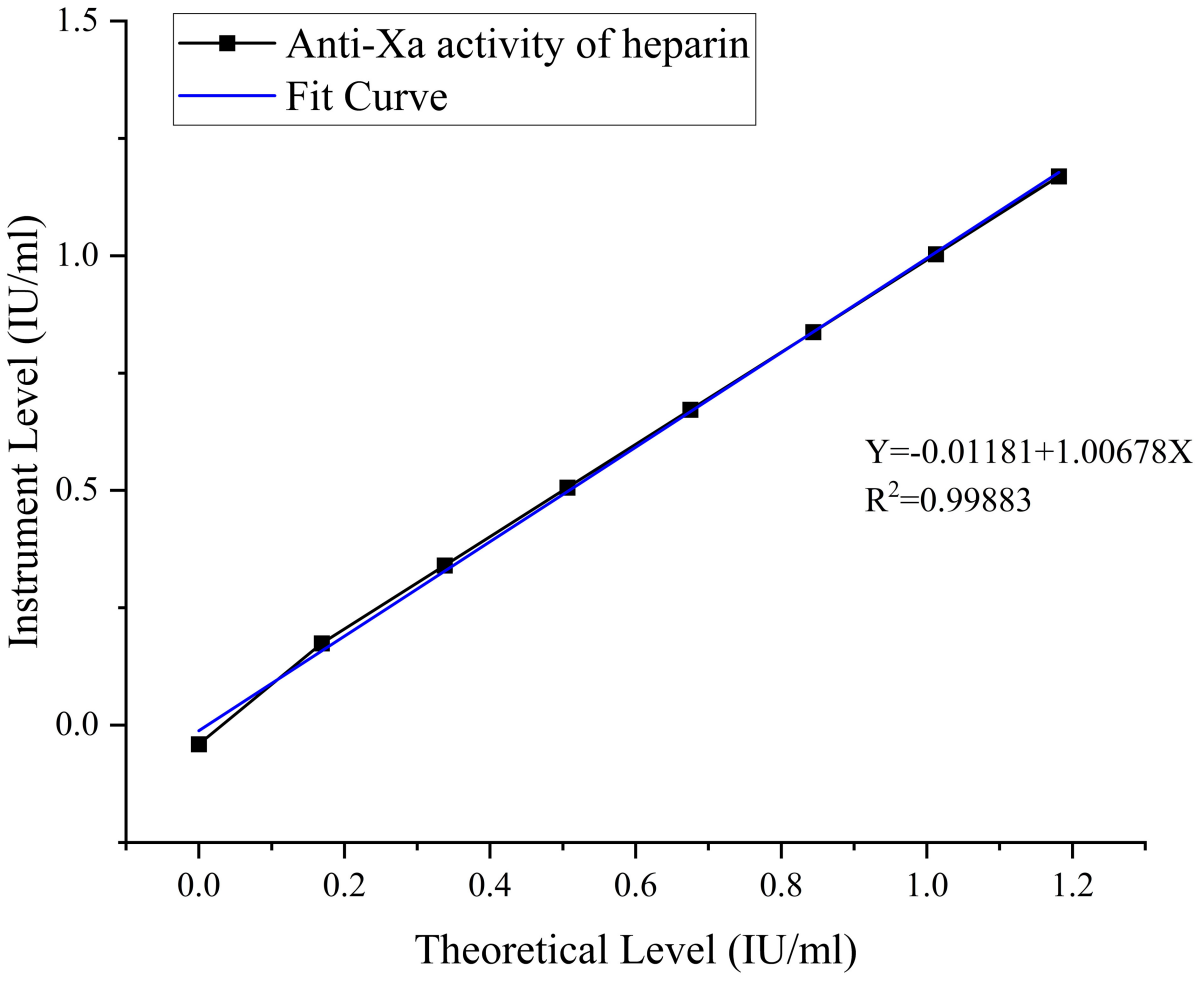
Standard curve for anti-Xa activity of heparin. Seven-point calibration curves using standard plasma (0–1.50 IU/ml). In-house system (*Y* = −0.01181 + 1.00678*X, R*^2^ = 0.99883).

In-house anti-factor Xa detection system: the calibration curve yielded an *R*^2^ value of 0.99883 with regression equation *Y* = −0.01181 + 1.00678*X* (Figure 3).

**Figure 4:**
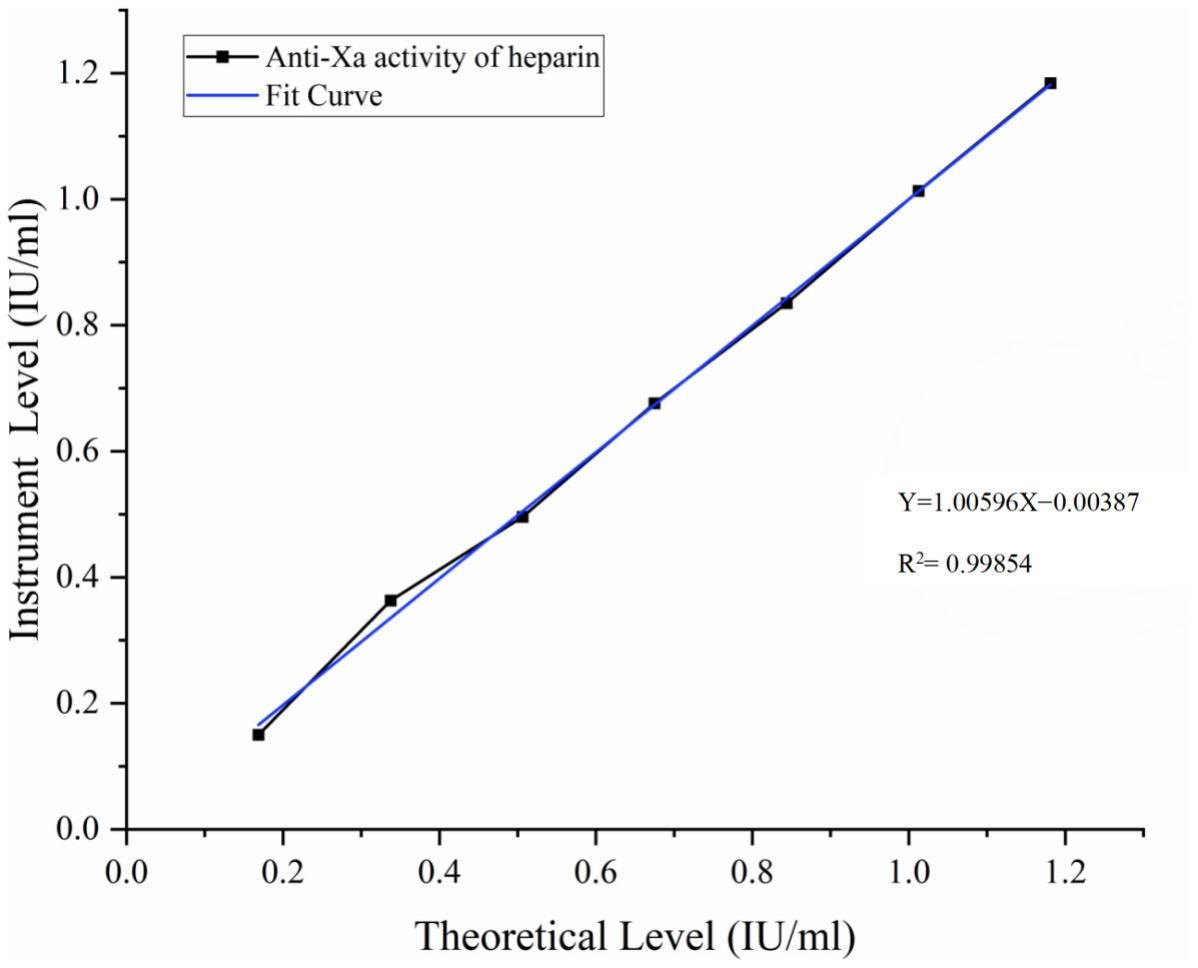
Standard curve for anti-Xa activity of heparin. Seven-point calibration curves using standard plasma (0–1.50 IU/ml). STA-Liquid system (*Y* = 7.1312 – 3.7510*X, R*^2^ = 0.99854). Each point represents duplicate measurements.

STA-Liquid Anti-Xa detection system: the calibration curve yielded an *R*^2^ value of 0.99854 with regression equationY =-0.00387 + 1.00596*X* (Figure 4).

Both systems demonstrated excellent linearity.

### 3.2 Analytical performance validation

#### 3.2.1 Precision study

We conducted precision studies at three quality control (QC) levels (0.30, 0.70, and 1.35 IU/ml) met CLSI acceptance performance. Intra-assay precision (coefficient of variation, CV) ranged from 1.15 to 4.32%, while Inter-assay precision (CV) ranged from 1.25 to 4.03%. All results met the acceptable criteria established by the Clinical and Laboratory Standards Institute (CLSI) EP05-A3 guideline (Table 1).

**Table 1:** Results of repeatability and intermediate precision.

**Precision type**	**QC level (IU/ml)**	**Mean (IU/ml)**	**SD (%)**	**CV (%)**
Intra-assay precision^a^	1.35	1.35	1.55	1.15
	0.70	0.71	1.79	2.52
	0.30	0.31	1.34	4.32
Inter-assay precision^b^	1.35	1.34	1.68	1.25
	0.70	0.70	1.44	2.06
	0.30	0.30	1.21	4.03

CV (%) = (SD/mean) × 100%. QC, quality control; SD, standard deviation; CV, coefficient of variation. ^a^Intra-assay precision: 20 consecutive measurements within a single run. ^b^Inter-assay precision: two runs per day over 20 days (n = 80).

#### 3.2.2 Analytical sensitivity

The limit of blank (LoB), limit of detection (LoD), and limit of quantitation (LoQ) were comprehensively evaluated according to CLSI guideline EP17-A2.

**Figure 5:**
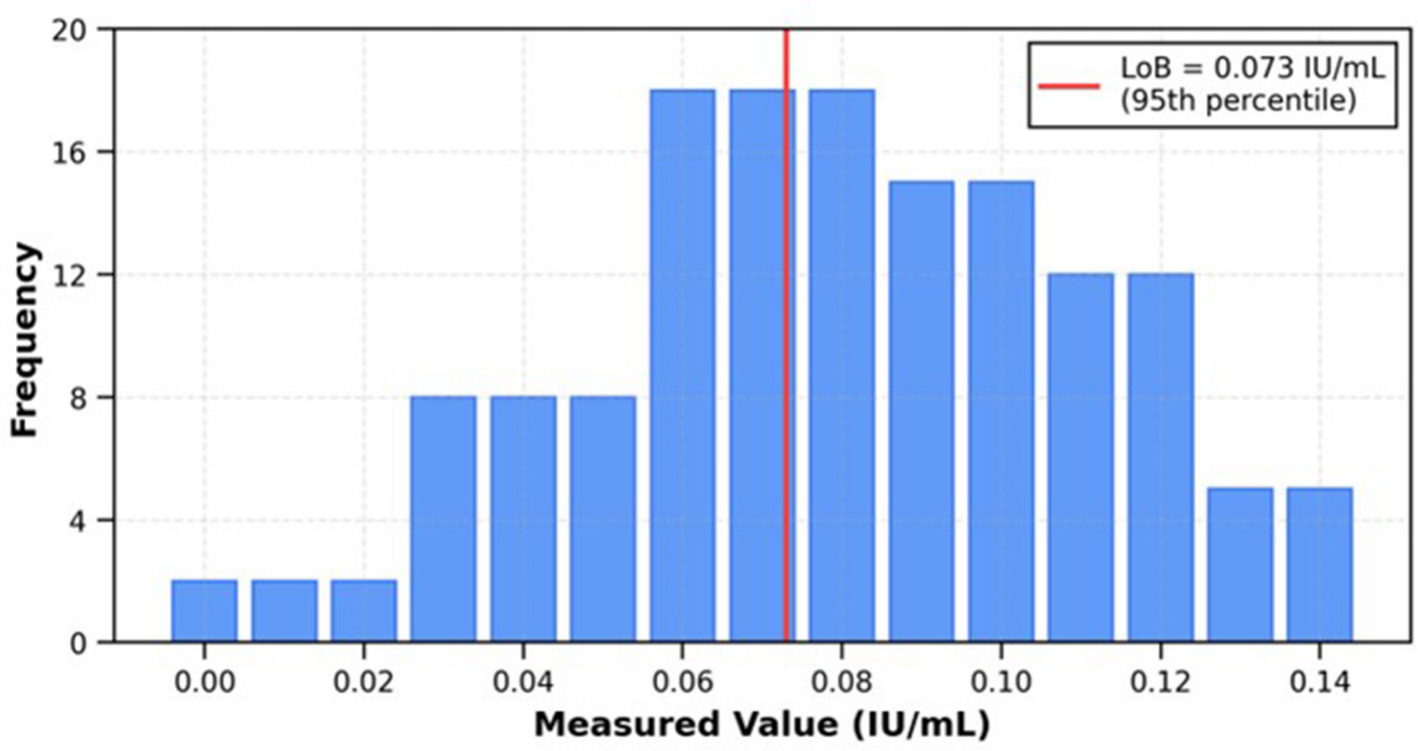
Analytical sensitivity evaluation. LoB determination showing distribution of 60 blank measurements (mean 0.042 IU/ml, LoB 0.073 IU/ml at 95th percentile).

Limit of blank (LoB): sixty blank measurements (normal pooled plasma with anti-Xa < 0.01 IU/ml) yielded a mean value of 0.042 ± 0.015 IU/ml. The LoB was established at 0.073 IU/ml (95th percentile; Figure 5).

Limit of detection (LoD): sixty measurements of low-concentration samples (target: 0.08 IU/ml, SD = 0.004 IU/ml) were performed. The LoD was calculated as 0.073 + 1.645 × 0.004 = 0.080 IU/ml.

Limit of quantitation (LoQ) was evaluated at three candidate concentrations (*n* = 20 each) based on three predefined criteria: median absolute deviation (MAD) ≤ 30%, probability of detection (POD) ≥95%, and total error (TE) ≤ 40%.

- At 0.04 IU/ml: MAD = 500%, POD = 42.1%, TE = 112.9% (failed all criteria).
- At 0.08 IU/ml: MAD = 28.57%, POD = 95.7%, TE = 43.4% (borderline performance).
- At 0.10 IU/ml: MAD = 20.0%, POD = 100%, TE = 39.2% (passed all criteria).

**Figure 6:**
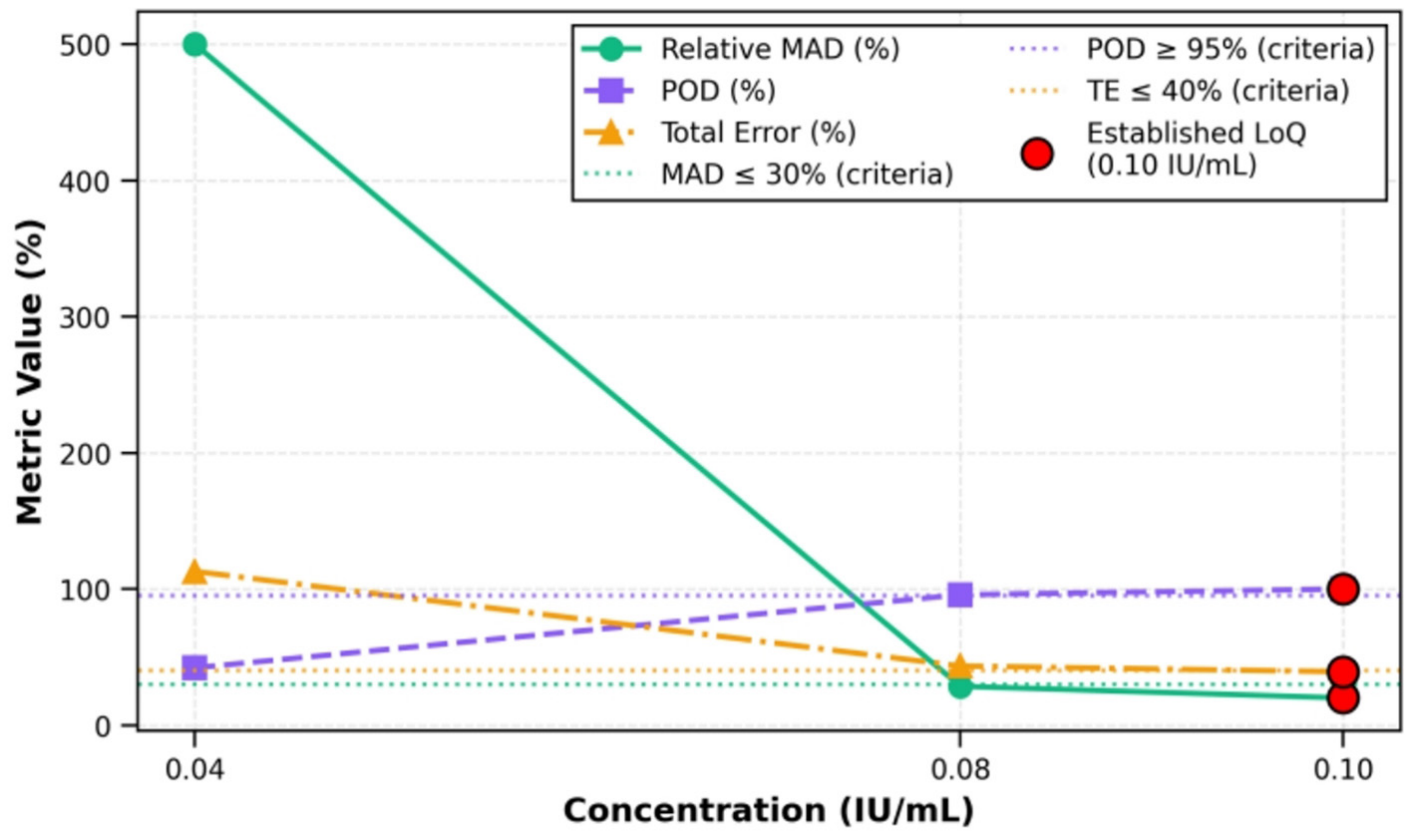
Analytical sensitivity evaluation. LoQ established at 0.10 IU/ml based on convergence of MAD ( ≤ 30%), POD (≥95%), and TE ( ≤ 40%) criteria. MAD, median absolute deviation; POD, probability of detection; TE, total error.

Therefore, we established the LoQ at 0.10 IU/ml (Figure 6).

#### 3.2.3 Linearity study

**Figure 7:**
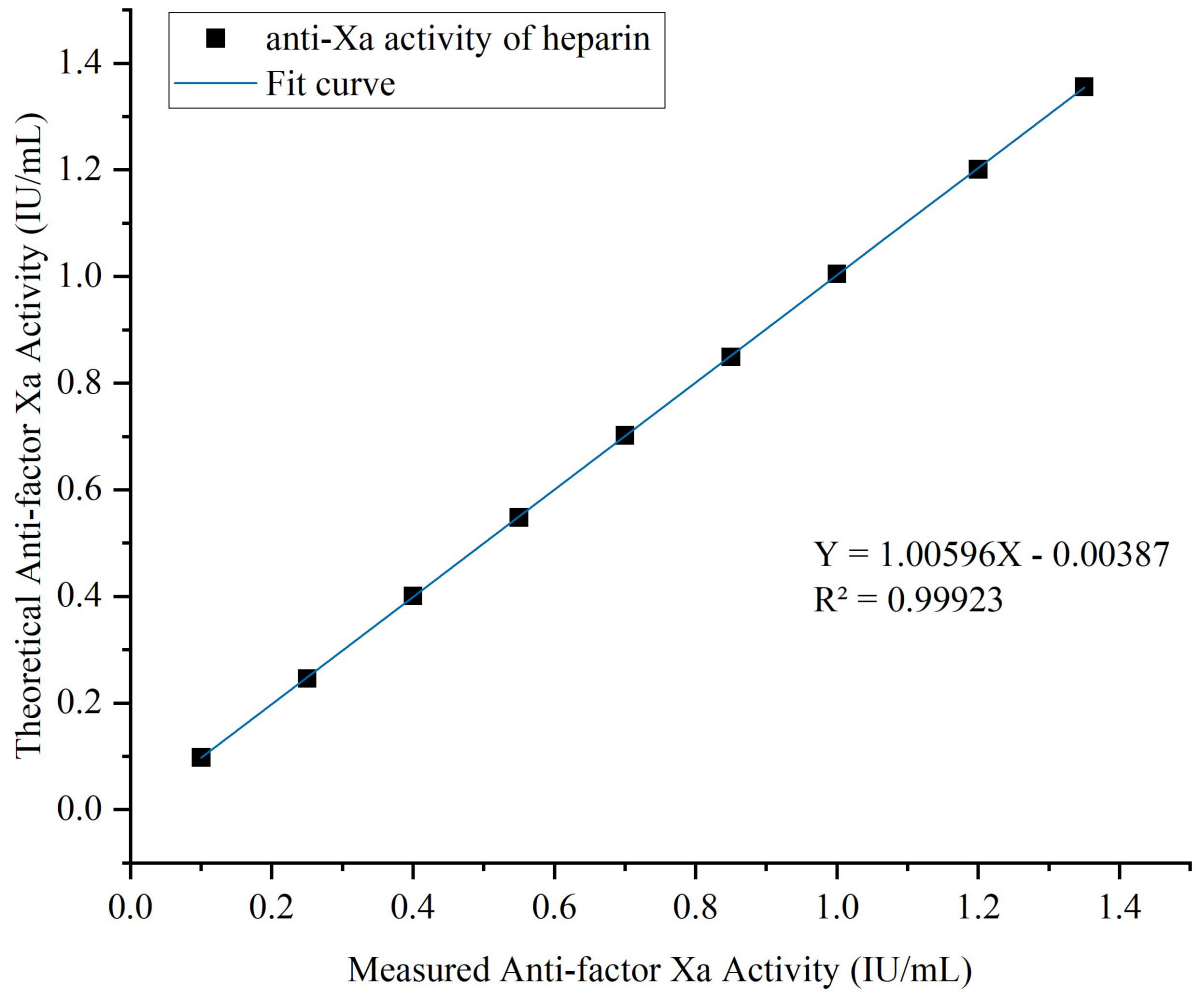
Linear range of the in-house anti-factor Xa detection system. Linear regression analysis (*Y* = 1.00596*X* – 0.00387, *R*^2^ = 0.99923) with duplicate measurements on three separate days (*n* = 6 per dilution level).

Linearity was evaluated at nine concentration points ranging from 0.10 to 1.35 IU/ml according to CLSI guideline EP06 (Figure 7). Linear regression demonstrated excellent linearity, with regression equation *Y* = 1.00596*X* – 0.00387 and coefficient of determination *R*^2^ = 0.99923.

#### 3.2.4 Carryover rate

The carryover rate was calculated using the formula: CR = |L1 – L3|/(H3 – L3) × 100% = |0.45 – 0.47|/(1.31 – 0.47) × 100% = 0.57%, which is well below the acceptance criterion of 3%, indicating negligible carryover contamination.

#### 3.2.5 Stability study

Stability of the complete liquid reagent system (R1: bovine factor Xa solution with BSA stabilizer; R2: S-2732 substrate solution with PEG-6000 stabilizer) was evaluated under thermal stress at 37 °C, for 14 days. The relative median absolute deviation (MAD) at all concentration levels remained ≤ 2.86%, meeting the CLSI EP25-A requirement of ≤ 10%. This indicates that reagent precision was unaffected and stability was satisfactory for routine clinical use.

### 3.3 Method comparison study

#### 3.3.1 Baseline characteristics and sample allocation

The derivation set (*n* = 80) and validation set (*n* = 110) showed no statistically significant differences in anti-Xa concentration distribution, age, or sex composition (*p* > 0.05), confirming successful random allocation.

#### 3.3.2 Correlation and agreement analysis

Eighty specimens stratified across the clinical range were analyzed by both the in-house and STA-Liquid (Stago) systems. Test results from both systems followed a normal distribution. Paired samples *t*-test showed no significant difference between the two detection systems (*p* > 0.05).

**Figure 8:**
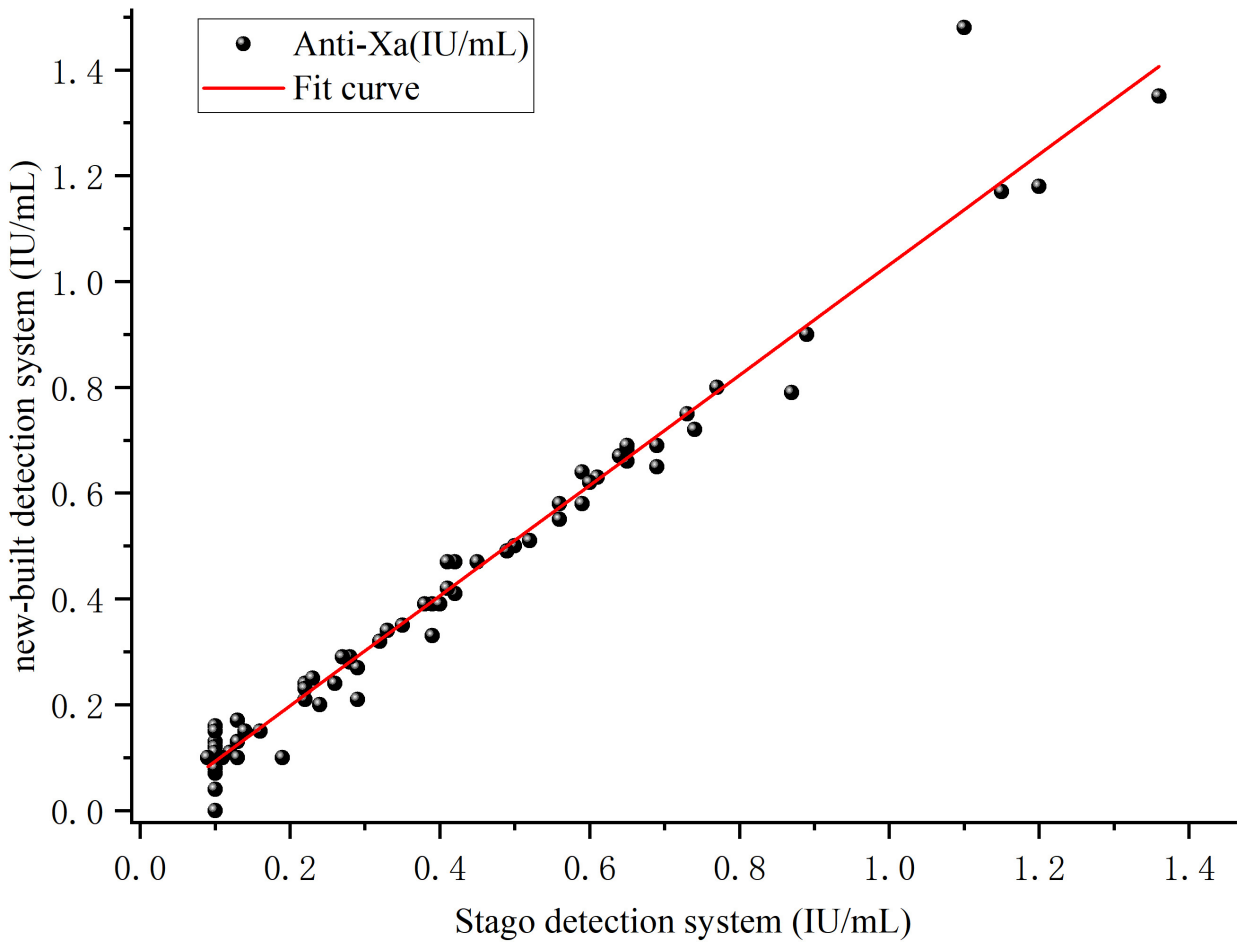
Method comparison between in-house and STA-Liquid anti-factor Xa systems. Correlation analysis showing strong linear relationship (*Y* = −0.01018 + 1.03984*X, R*^2^ = 0.972, Pearson *r* = 0.986, ρ < 0.001, *n* = 120).

Linear regression analysis yielded the equation *Y* = −0.01018 + 1.03984*X* with *R*^2^ = 0.972, and the Pearson correlation coefficient was *r* = 0.986 (*p* < 0.001; Figure 8).

**Figure 9:**
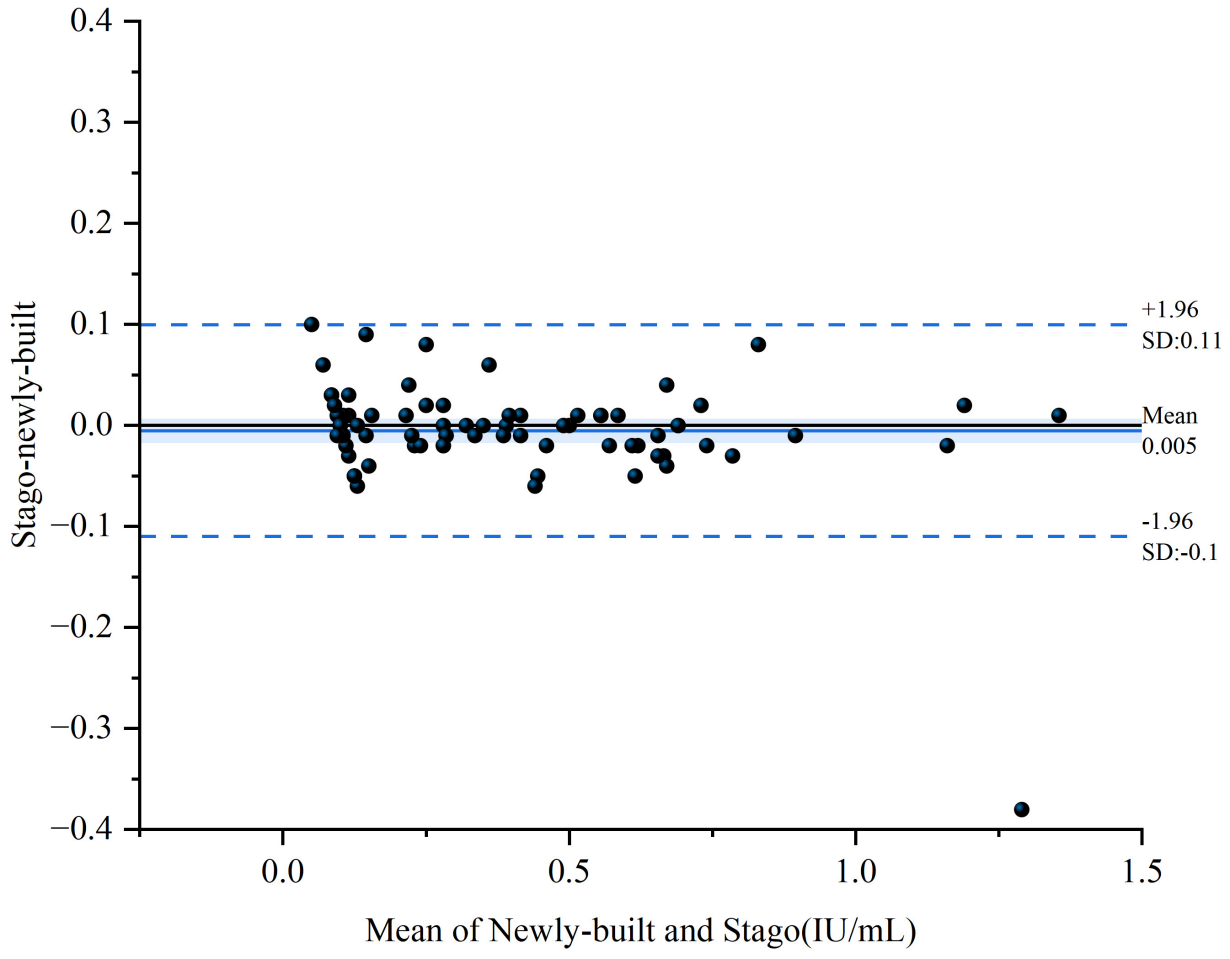
Method comparison between in-house and STA-Liquid anti-factor Xa systems. Bland-Altman analysis showing mean bias of 0.005 IU/ml with 95% limits of agreement at ±1.96 SD (−0.1 to 0.11 IU/ml); five points (6.25%) fell outside limits.

The Bland–Altman plot demonstrated 93.75% of samples within the two detection systems. Among 80 data points, only 5 (6.25%) fell outside the 95% limits of agreement, indicating excellent concordance (Figure 9).

Both sections used *n* = 80 derivation set but examined different variables. Section 3.3.2 tested anti-Xa values (normally distributed due to stratified sampling). Section 3.4.1 tested APTT values (non-normal distribution reflecting clinical clustering).

### 3.4 APTT therapeutic range calibrated against anti-factor Xa activity

#### 3.4.1 Correlation analysis

Since neither APTT nor anti-factor Xa activity followed normal distribution (Shapiro-Wilk test, *p* < 0.05), Spearman rank correlation analysis was employed. A moderate positive correlation was observed (Spearman's ρ = 0.678, *p* < 0.001). Linear regression yielded the equation *Y* = 132.7*X* + 44.1 (*R*^2^ = 0.46).

**Figure 10:**
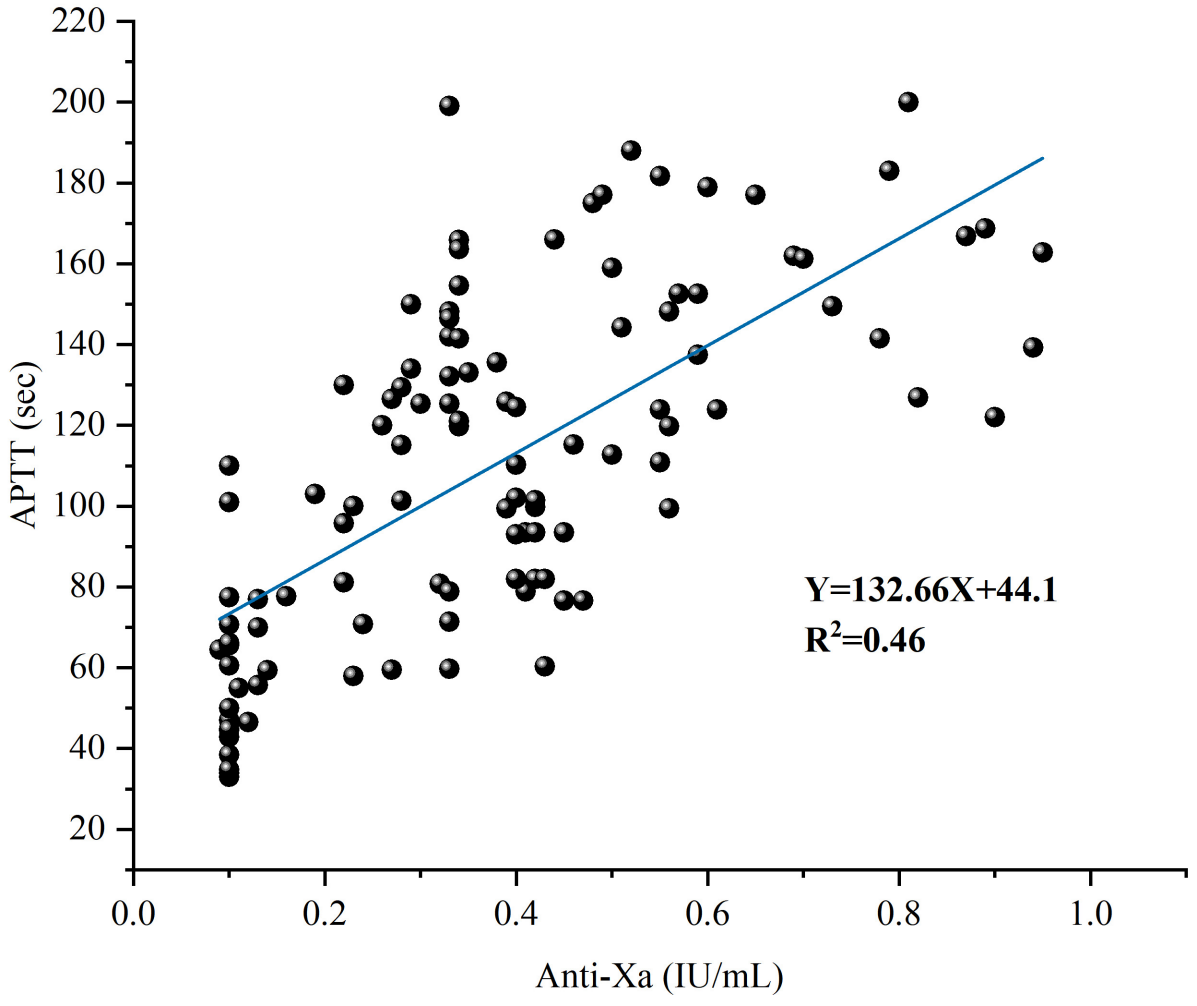
Correlation between APTT and anti-factor Xa activity. Linear regression analysis showing moderate correlation (*Y* = 132.7*X* + 44.1, *R*^2^ = 0.46, *n* = 110).

This moderate correlation indicates that only 46% of APTT variance is explained by anti-Xa activity, with the remaining 54% attributable to confounding factors including fibrinogen levels, factor VIII activity, and sample quality. This finding supports the need for careful interpretation when using APTT as a surrogate marker for anti-factor Xa monitoring (Figure 10).

#### 3.4.2 Linear regression method

Using the regression equation *Y* = 132.7*X* + 44.1, an anti-factor Xa level of 0.3 IU/ml corresponded to a predicted APTT value of 84 s, while 0.7 IU/ml corresponded to 137 s, yielding a preliminary range of 84–137 s.

#### 3.4.3 ROC curve analysis

**Figure 11:**
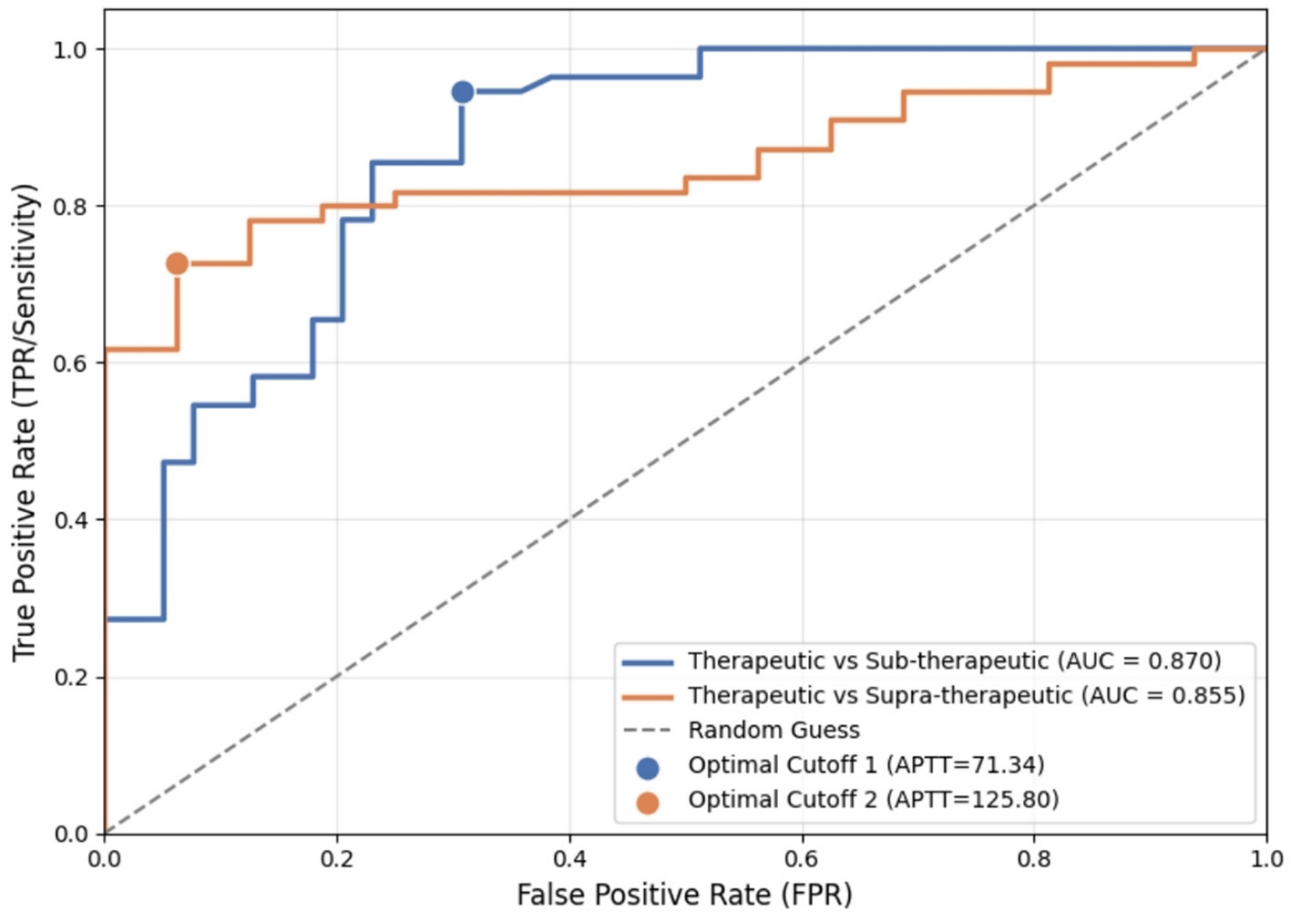
ROC curve analysis for APTT therapeutic threshold determination. ROC curves comparing therapeutic vs. sub-therapeutic (AUC = 0.870, optimal cutoff = 71.34 s) and therapeutic vs. supra-therapeutic (AUC = 0.855, optimal cutoff = 125.80 s).

Receiver operating characteristic (ROC) analysis identified optimal APTT cutoff values for distinguishing therapeutic from non-therapeutic anticoagulation levels (Figure 11).

Lower threshold (0.3 IU/ml): for distinguishing therapeutic levels (≥0.3 IU/ml) from subtherapeutic levels (< 0.3 IU/ml), the optimal APTT cutoff was 78.9 s. Performance metrics: AUC = 0.723, sensitivity 70.6%, specificity 71.6%.

Upper threshold (0.7 IU/ml): for distinguishing therapeutic levels ( ≤ 0.7 IU/ml) from supratherapeutic levels (>0.7 IU/ml), the optimal APTT cutoff was 126.6 s. Performance metrics: AUC = 0.815, sensitivity 83.3%, specificity 80.0%.

ROC-derived cutoffs were slightly lower than regression-predicted values, reflecting the balance between sensitivity and specificity. Based on integrating both methods and prioritizing clinical safety, a laboratory-specific APTT therapeutic range of 79–127 s was established, corresponding to anti-factor Xa 0.3–0.7 IU/ml.

#### 3.4.4 Final therapeutic range

A laboratory-specific APTT therapeutic range of 79–127 s was established, corresponding to anti-factor Xa levels of 0.3–0.7 IU/ml, Although linear regression predicted 84–137 s, ROC-derived cutoffs (78.9 and 126.6 s) were adopted due to superior diagnostic accuracy (AUC 0.723–0.815) with balanced sensitivity (70.6%−83.3%) and specificity (71.6%−80.0%), ensuring optimal clinical decision-making for UFH monitoring.

A laboratory-specific APTT therapeutic range of 79–127 s was established, corresponding to anti-factor Xa levels of 0.3–0.7 IU/ml. Compared with the traditional 1.5–2.5 × baseline method (54–90 s), which resulted in a 42.7% misclassification rate, the ROC-optimized range reduced misclassification to 25.5%. Notably, the traditional method led to underdosing risk, as 54% of patients with APTT values of 54–79 s were actually subtherapeutic, while 38% of patients with APTT >90 s were within the therapeutic range, raising false overdosing concerns. Although linear regression predicted a range of 84–137 s, ROC-derived cutoffs (78.9 and 126.6 s) were adopted due to superior diagnostic accuracy (AUC 0.723–0.815) with balanced sensitivity (70.6%−83.3%) and specificity (71.6%−80.0%). These findings support the CLSI H21-A5 recommendation for laboratory-specific therapeutic range establishment, ensuring optimal clinical decision-making for UFH monitoring.

### 3.5 Classification concordance analysis

#### 3.5.1 Concordance results

**Figure 12:**
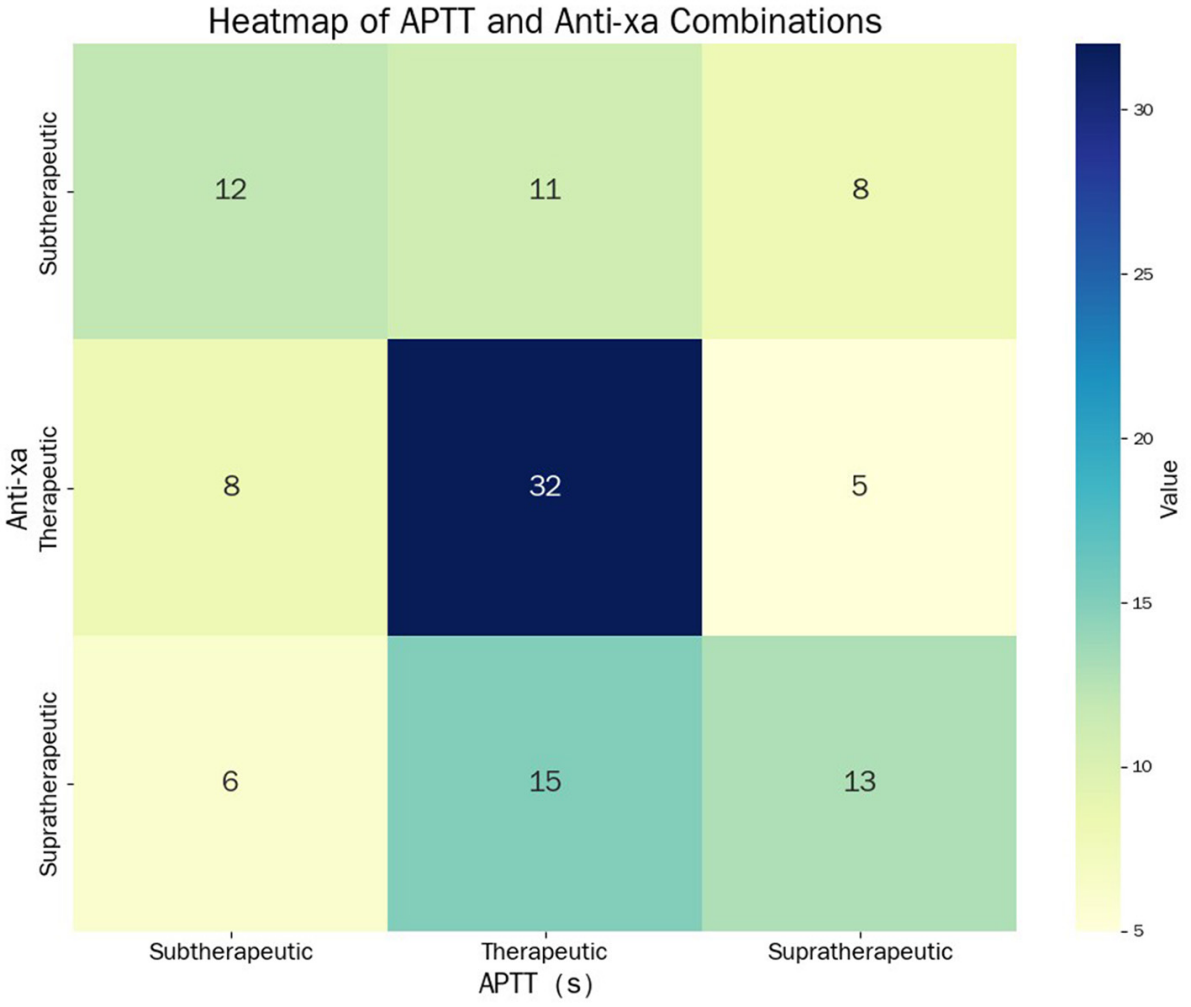
Concordance analysis between APTT and anti-factor Xa classifications. Heatmap showing distribution of 110 patients across APTT and anti-Xa categories with concordance and discordance patterns.

Among 110 paired measurements, 46 pairs (41.8%) showed concordant classification between APTT and anti-factor Xa, while 64 pairs (58.2%) were discordant (Figure 12). The concordant pairs comprised 8 subtherapeutic (7.3%), 32 therapeutic (29.1%), and 6 supratherapeutic (5.5%) classifications.

#### 3.5.2 Discordance by clinical indication

UFH indications and indication-specific discordance rates are summarized in Table 2. ICU/surgical populations demonstrated numerically higher discordance rates (73.3%−75.0%) compared to medical indications (52.6%−54.2%), though this difference did not reach statistical significance (*p* = 0.156) due to limited subgroup sizes. This trend suggests that critically ill patients may be particularly susceptible to APTT-based monitoring inaccuracies due to higher prevalence of confounding factors including elevated fibrinogen, acute phase responses, and renal dysfunction.

**Table 2:** UFH indications and APTT/anti-factor Xa classification discordance rates.

**Indication**	***n* (%)**	**Discordance rate**
Acute coronary syndrome	38 (34.5)	52.6%
VTE treatment/prophylaxis	24 (21.8)	54.2%
Post-cardiac surgery	15 (13.6)	73.3%
Hemodialysis/CRRT	8 (7.3)	75.0%
Other	25 (22.7)	56.0%

Discordance was defined as different therapeutic classification between APTT-based (79–127 s) and anti-factor a-based (0.3–0.7 IU/ml) methods. “Other” includes arterial thrombosis and bridging therapy. ICU/surgical indications (73.3%−75.0%) vs. medical indications (52.6%−54.2%), p = 0.156. UFH, unfractionated heparin; VTE, venous thromboembolism; CRRT, continuous renal replacement therapy.

### 3.6 Predictive model for detection discordance

#### 3.6.1 Random forest model

**Figure 13:**
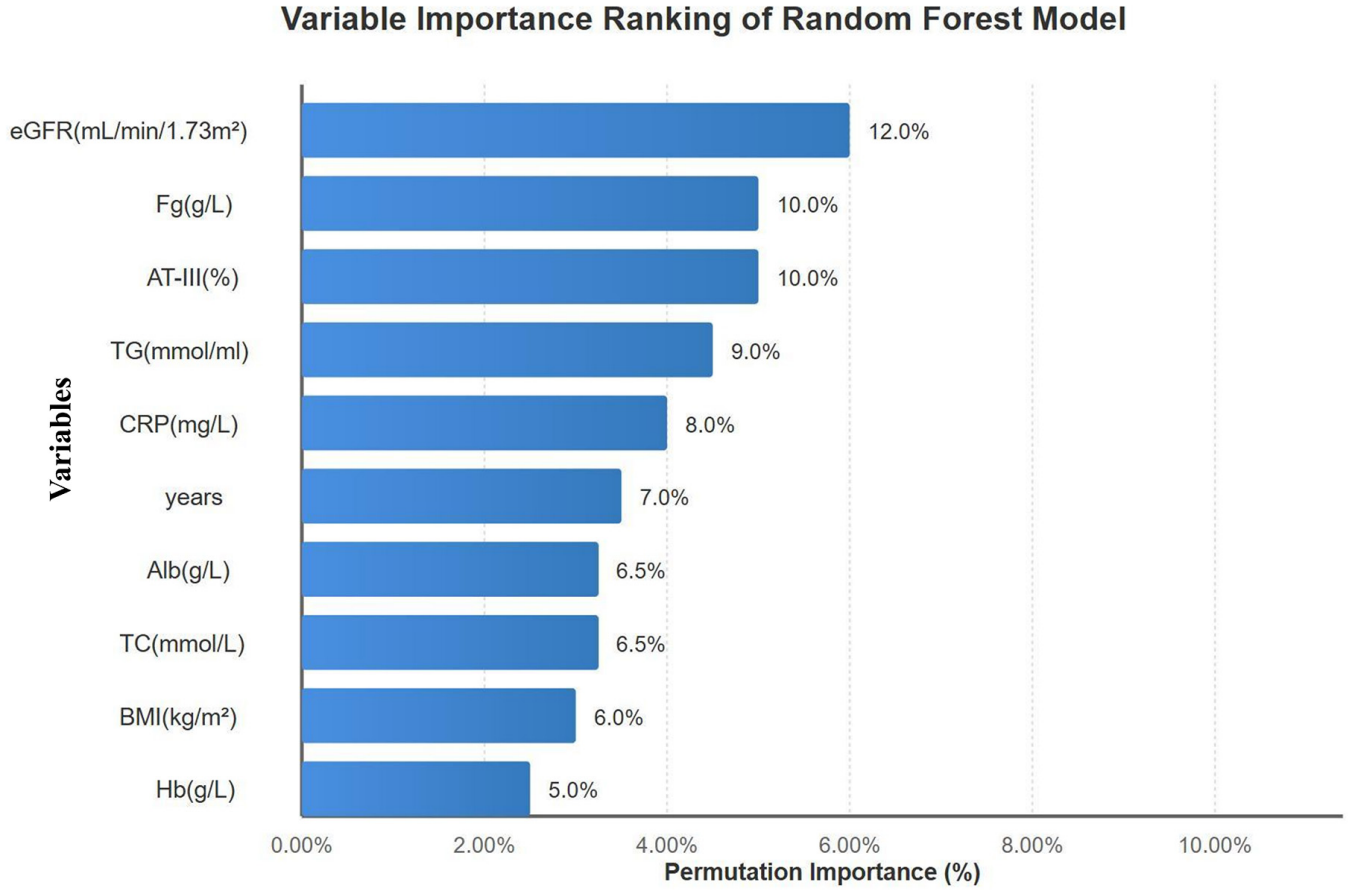
Variable importance ranking of the random forest model. Permutation importance scores showing eGFR (12.0%), fibrinogen (10.0%), and AT-III (10.0%) as top predictors. Error bars represent standard deviation from 5-fold cross-validation.

A random forest model incorporating 10 clinical and laboratory variables was constructed to predict concordance vs. discordance between APTT and anti-factor Xa measurements. Given the limited sample size, 5-fold cross-validation was employed to assess model performance. Results showed a mean AUC of 0.79 (95% CI: 0.71–0.87), accuracy of 78.8%, sensitivity of 64.3%, and specificity of 89.5%. Permutation importance analysis ranked the 10 variables in descending order of importance: eGFR, Fibrinogen (Fg), AT-III, TG, CRP, age, ALB, TC, BMI, and Hb (Figure 13). All predictive features demonstrated good stability across 5-fold cross-validation (coefficient of variation CV < 1.0).

#### 3.6.2 Logistic regression model

Ten variables were entered into multivariable logistic regression based on literature evidence and clinical relevance. Backward stepwise regression identified five independent predictors: Fg (OR = 3.67, 95% CI: 1.67–8.07, *p* = 0.001), TG (OR = 1.50, 95% CI: 1.08–2.08, *p* = 0.015), eGFR (OR = 1.03, 95% CI: 1.00–1.05, *p* = 0.025), CRP (OR = 1.07, 95% CI: 1.00–1.14, *p* = 0.041), and AT-III (OR = 1.015, 95% CI: 1.002–1.028, *p* = 0.023). The model achieved an AUC of 0.726 (95% CI: 0.638–0.814), with accuracy of 68.2%, sensitivity of 73.1%, and specificity of 63.8%. Multicollinearity diagnostics showed all variables had variance inflation factor (VIF) < 2.0 (mean VIF = 1.23), and the Hosmer–Lemeshow goodness-of-fit test yielded satisfactory results (χ^2^ = 6.823, *p* = 0.555).

#### 3.6.3 Consistency analysis between the two models

Both models demonstrated high consistency in identifying core predictive factors. The five key variables commonly identified (Fg, AT-III, eGFR, TG, CRP) ranked among the top features in both models. The random forest model exhibited superior overall predictive performance (AUC = 0.79) and specificity (89.5%), while the logistic regression model showed slightly better sensitivity (73.1%). The complementary nature of these two models provides comprehensive reference for clinical decision-making.

## 4 Discussion

UFH remains a cornerstone anticoagulant in critical care settings worldwide. However, optimal therapeutic monitoring continues to pose significant clinical challenges. While APTT has served as the conventional monitoring method for over five decades, accumulating evidence reveals substantial limitations including high inter-laboratory variability due to significant differences in reagent sensitivity (17, 33), susceptibility to pre-analytical factors, and poor correlation with clinical outcomes (34). The 2012 American College of Chest Physicians guidelines acknowledge these limitations and recommend anti-factor Xa assays as an alternative, particularly in patients with baseline coagulation abnormalities (26).

Despite these recommendations, anti-factor Xa assay adoption remains limited in clinical practice. Recent surveys indicate that only approximately one-quarter of institutions routinely employ anti-factor Xa monitoring, with cost frequently cited as a primary barrier A Swiss prospective study reported total costs of 24.15 Swiss Francs (approximately $25 USD) for anti-Xa vs. 23.40 Swiss Francs for APTT per test (14, 35). This cost disparity creates economic barriers particularly in resource-limited settings. Furthermore, monitoring method discordance occurs in 30%−50% of cases, creating clinical uncertainty and potentially inappropriate dosing adjustments. This discordance disproportionately affects high-risk populations including ICU patients, those with acute phase reactions, and individuals with renal dysfunction (36, 37).

To address these barriers, we developed and validated an in-house chromogenic anti-factor Xa assay optimized for routine clinical implementation. Through systematic concentration gradient experiments, optimal reagent compositions were identified, achieving balanced sensitivity, linearity, and stability. The incorporation of BSA (0.1%−0.5% w/v) as an R1 stabilizer and PEG-6000 (0.1%−0.5% w/v) for R2 extends reagent shelf-life to 14 days at 37 °C with relative MAD ≤ 2.86%, reducing waste compared to commercial systems that require daily preparation. The optimized sample-to-reagent ratio requires only 5 μl of patient plasma per test, substantially reducing sample volume requirements compared to commercial platforms. This miniaturization proves particularly valuable in neonatal, pediatric, and critically ill populations where sample volume constraints are significant (38). The in-house monitoring system completes analysis within 5 min, facilitating rapid clinical decision-making. Preliminary cost analysis indicated reagent costs of approximately $2.00 per test, including calibrators, quality controls, and consumables, representing a substantial cost reduction compared to commercial systems.

Our higher discordance rate (58.2% vs. 30%−50%) reflects: (1) a high-risk population—post-cardiac surgery (13.6%) and hemodialysis/CRRT (7.3%) patients showed 73%−75% discordance; (2) ROC-optimized rather than empirical APTT ranges, revealing true biological discordance; and (3) stricter three-category classification. This high discordance actually validates anti-Xa utility in populations where accurate monitoring is most critical.

Rigorous validation following CLSI guidelines demonstrated analytical performance meeting international standards across all parameters. Intra-assay CV of 1.15%−4.32% and inter-assay CV of 1.25%−4.03% across the therapeutic range (0.30–1.35 IU/ml) compare favorably with reported commercial system performance (39, 40). Excellent linearity across 0.10–1.35 IU/ml (*R*^2^ = 0.999, recovery 90%−110%) exceeds CLSI acceptability criteria. Direct comparison with the Stago STA-Liquid Anti-Xa platform demonstrated strong correlation (Pearson *r* = 0.986, *R*^2^ = 0.972) with 93.75% of samples within Bland-Altman limits of agreement and mean bias of −0.008 IU/ml. These results demonstrate that our in-house platform provides results comparable to the commercial reference method. Carryover rate of 0.57% and 14-day thermal stability ensure reliable performance in routine clinical workflows.

To evaluate clinical performance, we applied the newly established anti-factor Xa assay to UFH monitoring in hospitalized patients. Our clinical validation findings provide evidence supporting anti-factor Xa measurement over APTT-based monitoring. Although APTT and anti-factor Xa demonstrated moderate correlation (Spearman ρ = 0.678, *p* < 0.001), classification agreement at clinically relevant thresholds was poor (41.8%, Cohen's κ = 0.242), with 58.2% discordance rate consistent with prior reports (41–43). This substantial discordance persisted despite establishing laboratory-specific APTT therapeutic ranges (79–127 s) through ROC analysis. It is worth noting that optimized APTT cutoffs achieved only moderate diagnostic accuracy (AUC 0.723–0.815, sensitivity 70.6%−83.3%, specificity 71.6%−80.0%), indicating that approximately 20%−30% of patients would be misclassified even with laboratory-specific calibration. These findings underscore the limitations of APTT-based UFH monitoring and support the utility of direct anti-factor Xa measurement, particularly in high-risk populations.

We applied machine learning approaches to identify clinical and laboratory predictors of APTT-anti-factor Xa discordance. Random forest modeling (AUC = 0.79, 95% CI: 0.71–0.87; specificity 89.5%) ranked eGFR, fibrinogen, and AT-III as important predictive features. Logistic regression analysis (sensitivity 73.1%, AUC = 0.726) identified five independent predictors: fibrinogen (OR = 3.67, 95% CI: 1.67–8.07), triglycerides (OR = 1.50, 95% CI: 1.08–2.08), eGFR (OR = 1.03, 95% CI: 1.00–1.05), CRP (OR = 1.07, 95% CI: 1.00–1.14), and AT-III (OR = 1.015, 95% CI: 1.002–1.028), with acceptable multicollinearity (all VIF < 2.0). These findings align with known pathophysiologic mechanisms wherein elevated fibrinogen and acute phase reactants prolong APTT independent of heparin effect, hypertriglyceridemia causes spurious APTT prolongation through sample turbidity, renal dysfunction affects heparin clearance, and AT-III variability directly impacts heparin anticoagulant activity (33, 44–47).

**Figure 14:**
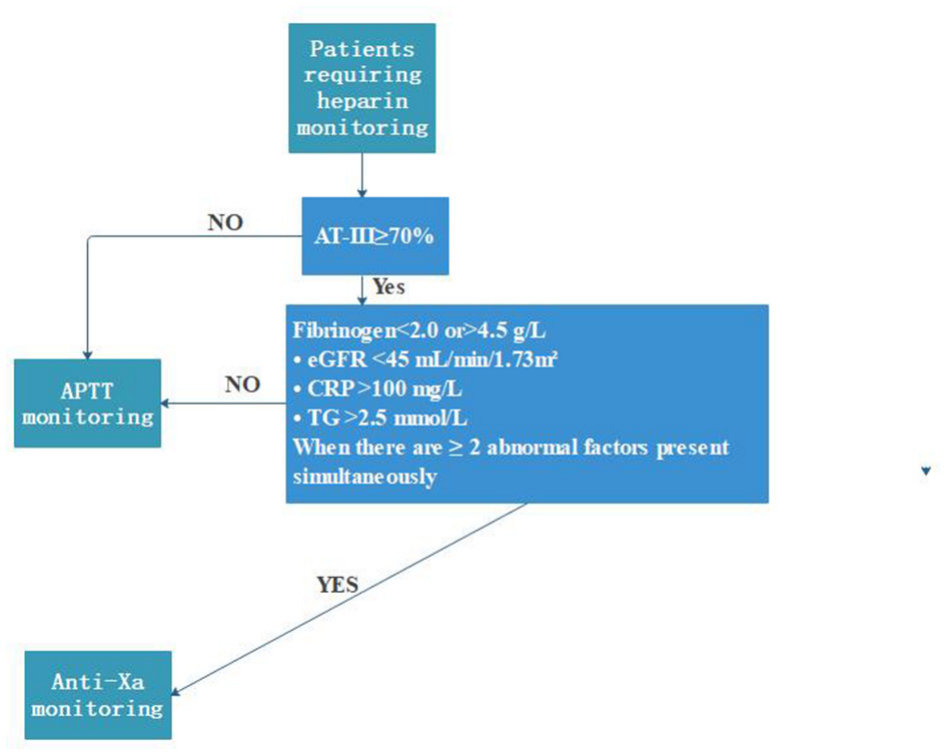
Clinical algorithm for heparin monitoring strategy selection. Decision flowchart based on AT-III level (≥70% for APTT; < 70% with high-risk criteria for anti-factor Xa monitoring). High-risk criteria include fibrinogen ≤ 2.0 or ≤ 4.5 g/L with additional abnormalities (eGFR < 45 ml/min/1.73 m^2^, albumin ≤ 17 g/L, CRP >45 mg/L, TG ≥2.5 mmol/L, or ≥2 factors simultaneously).

Based on these findings and existing literature (19, 48, 49), we present a preliminary, hypothesis-generating framework (Figure 14). This framework is not intended to guide clinical practice without prospective validation with clinical endpoints (bleeding/thrombosis events; Figure 14). This framework suggests preferential anti-factor Xa monitoring for patients with fibrinogen ≤ 2.0 or ≤ 4.5 g/L plus additional abnormalities including eGFR ≥45 ml/min/1.73 m^2^, albumin ≤ 17 g/L, CRP >45 mg/L, TG ≥2.5 mmol/L, or concurrent presence of multiple factors. For patients without these predictors, APTT monitoring may remain adequate. However, due to modest sample size (*n* = 110, with approximately 64 discordant events yielding events-per-variable < 7), this framework represents exploratory guidance requiring validation in larger multicenter studies. Whether addressing these discordances through selective anti-factor Xa monitoring improves patient outcomes remains uncertain and requires evaluation in prospective trials. We emphasize that this framework should not guide clinical practice without rigorous prospective validation.

The anti-factor Xa assay established in this study demonstrates several key advantages that support its clinical implementation. The assay exhibits excellent analytical performance with good linearity across its measurement range (Pearson *r* = 0.986, *R*^2^ = 0.972) and precision that meets international quality standards (intra-assay CV ≤ 4.32%, inter-assay CV ≤ 4.03%). The chromogenic substrate method provides superior stability and precision compared with traditional coagulation-based detection kits. Clinical validation against the Stago commercial platform confirmed high correlation and consistency when detecting anti-factor Xa activity in patients receiving UFH therapy, establishing the assay's reliability for therapeutic monitoring. Notably, the assay offers significant practical advantages including rapid turnaround time with complete detection within 5 min and substantially reduced cost at approximately $2.0 per test, which is considerably lower than commercial platforms and may facilitate broader clinical adoption of anti-factor Xa testing in resource-limited settings.

However, this study also has several limitations: (1) Limited sample size—single-center study (*n* = 110, 64 events), risking overfitting and institution-specific results (50, 51); (2) Cross-sectional design, unable to assess dynamic changes; (3) Absence of bleeding/thrombotic event data, precluding outcome verification; and (4) Only internal cross-validation, lacking external validation.

The next steps would be to explore optimal lyophilization parameters to extend reagent shelf-life and facilitate broader distribution. Future research should prioritize multicenter external validation across diverse clinical settings to confirm assay performance and predictive model transportability. Prospective studies linking monitoring discordances to clinical outcomes including bleeding, thrombosis, and mortality are needed. Studies adequately powered for special populations such as ICU and critically ill patients are needed to define population-specific monitoring strategies. Formal cost-effectiveness analysis incorporating clinical outcomes and resource utilization should be conducted. In addition, increasing the sample size to establish more detailed reference intervals according to age and sex would improve clinical applicability.

Future research should focus on: (1) Multicenter external validation (target *n* ≥ 300) across diverse clinical settings to enhance generalizability; (2) Prospective cohort studies linking monitoring discordances between anti-Xa and APTT to clinical outcomes, specifically bleeding and thrombotic events; (3) Randomized controlled trials comparing anti-Xa-guided vs. APTT-guided anticoagulation strategies to determine optimal monitoring approaches; (4) Adequately powered studies in special populations, including intensive care unit patients, obstetric patients, and oncology patients, to address population-specific anticoagulation challenges; (5) Cost-effectiveness analysis evaluating the economic impact of different monitoring strategies in clinical practice; and (6) Dynamic monitoring protocols examine temporal changes in inflammatory markers, renal function, and coagulation factors to better understand the evolution of anticoagulation responses over time.

## 5 Conclusion

This study developed an in-house anti-factor Xa assay with analytical performance comparable to commercial systems. Poor categorical agreement between APTT and anti-factor Xa highlights APTT limitations for UFH monitoring. Ten clinical and laboratory factors potentially associated with monitoring discordance were identified, and a laboratory-specific APTT range (79–127 s) was derived.

However, these findings are substantially limited by single-center design, small sample size (*n* = 110), lack of external validation, and absence of clinical outcome data. The predictive models are hypothesis-generating and should not guide clinical practice without rigorous prospective validation. Multicenter studies with adequate sample sizes and clinical endpoint assessment are essential to determine whether these findings translate to improved patient outcomes.

## Data Availability

The original contributions presented in the study are included in the article/supplementary material, further inquiries can be directed to the corresponding author.
